# The extent of kidney involvement in paediatric tuberous sclerosis complex

**DOI:** 10.1007/s00467-024-06417-2

**Published:** 2024-06-04

**Authors:** Andrew Limavady, Matko Marlais

**Affiliations:** 1 Great Ormond Street Institute of Child Health, University College London, London, UK; 2 Department of Paediatric Nephrology, Great Ormond Street Hospital, London, UK

**Keywords:** Tuberous sclerosis complex, Paediatric, Kidney disease, Survival analysis

## Abstract

**Background:**

Tuberous sclerosis (TSC)–associated kidney disease is a leading cause of mortality in adults with TSC. This study aimed to understand TSC features in children, particularly kidney involvement, to inform clinical care for this specific group.

**Methods:**

This retrospective cohort study included all paediatric (< 19 years) TSC cases at a large tertiary paediatric nephrology centre. Relevant data were collected from patients’ records, statistical analyses were performed to identify associations between variables, survival probabilities were estimated with Kaplan‒Meier curves, and log-rank tests were conducted to assess survival differences among genetic mutations.

**Results:**

A total of 182 children with TSC were included. Among the 145 children with available kidney imaging data, 78.6% (114/145) exhibited kidney lesions. Angiomyolipomas (AMLs) were significantly more prevalent in the *TSC2* mutation group (*p* = 0.018). Children with *TSC2* mutations generally had poorer lesion-free survival than those with *TSC1* mutations, but this difference was only significant for AMLs (*p* = 0.030). The change in size of largest AMLs increased with age and doubled in children above 9 years; a similar pattern was observed when stratified by genetic mutation. In contrast, kidney cysts exhibited two peaks: one in children under 5 years (2.31 mm/year) and the second in children between 15–19 years (2.82 mm/year). Chronic kidney disease was observed in 12.3% (10/81) of children, and high-risk AMLs above 3 cm were observed in 9% (13/145).

**Conclusions:**

While TSC kidney disease emerges later in the disease course than neurological features, our findings emphasise the importance of kidney surveillance during childhood, including routine kidney imaging, kidney function, and blood pressure monitoring.

**Graphical abstract:**

**Figure:**
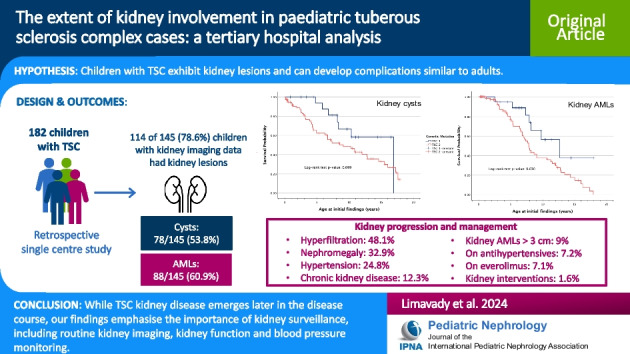
A higher resolution version of the Graphical abstract is available as Supplementary information

**Supplementary Information:**

The online version contains supplementary material available at 10.1007/s00467-024-06417-2.

## Introduction

Tuberous sclerosis complex (TSC) is an autosomal dominant condition characterised by typical skin lesions and benign tumour (hamartoma) development in multiple organs, including the brain, heart, and kidney. Mutations in *TSC1* and/or *TSC2* genes, encoding for hamartin and tuberin respectively, have been implicated in this disease, disrupting the mechanistic target of the rapamycin pathway, and consequently promoting tumour formation across organs [1, 2].

Despite TSC being attributed to two genes, over 4500 pathogenic or likely pathogenic variants in *TSC1* and over 13,000 in *TSC2* are known, leading to challenging molecular diagnoses [3] and diverse organ manifestations. Notably, these manifestations have an age-dependent expression. Cardiac rhabdomyomas and hypomelanotic macules are observed in over 50% of TSC newborns, while kidney lesions are found later [4]. TSC kidney disease often presents in three forms—angiomyolipoma (AML), cystic kidney disease, and renal cell carcinoma (RCC)—with varying prevalence reported across studies. A recent review by Bissler et al. [5] indicated that 70–80% of TSC patients have AMLs, approximately 50% have cysts, and less than 2% have RCC. Although TSC kidney disease emerges later in childhood and increases with age [5], it is the leading cause of mortality in adults [6]; thus, childhood presents a window of opportunity to initiate kidney prevention strategies and avert progression to chronic kidney disease (CKD), hypertension, and fatal haemorrhage [7, 8].

Most available literature on the incidence and disease characteristics of TSC, including kidney involvement, has mainly included adult patients. Only a few studies were done exclusively in children [9–11], though the TOSCA (TuberOus SClerosis registry to increase disease Awareness) study included over 1300 children out of 2065 patients with TSC in their analysis [12]. With the scarcity of evidence available in children, the management guidelines and recommendations for TSC-associated kidney disease in this population have largely been from the findings of limited studies. This study aims to contribute additional evidence and enhance our understanding of TSC features in children, with a particular focus on kidney involvement, aimed at advancing care for this specific demographic. Additionally, considering the risk of acute haemorrhage and CKD, this study aims to elucidate disease progression and assess the risk of kidney complications by genotype, with the goal of promoting improved surveillance practices.

## Methods

This retrospective cohort study was conducted at Great Ormond Street Hospital for Children in London, including all patients with genetic or clinical TSC diagnosis under 19 years of age at the time of diagnosis and who received care between January 2011 and December 2022. Any patient with insufficient clinical information in the electronic patient records was excluded. Formal ethical review was not needed; this study was conducted as part of a registered clinical audit.

Patient data, including demographic information, clinical symptoms, comorbidities, blood pressure readings, genetics, laboratory results, and clinical outcomes, were recorded. Kidney imaging data were obtained from radiology reports, noting the examination date, the type of imaging (ultrasound, MRI, or CT), kidney length, and the presence of cysts, AMLs, and RCC, along with their number, size, and location. Only the diameter of the largest cyst and AML was recorded. Any AML, whether containing macroscopic fat or fat-poor, was noted, though the latter would be excluded from subsequent analyses of AML. Data were collected at the initial presentation and at annual time points where longitudinal data were available. The change in the size of kidney lesions, encompassing both cysts and AMLs, was examined by quantifying the differences in the measurements of the largest kidney lesion, divided by the duration between successive imaging sessions. In instances where multiple sizeable lesions were identified in imaging reports, measurements from lesions occupying corresponding locations (e.g. right upper pole, left lower pole) were noted. These rates were further stratified based on the age groups of patients at the time of imaging. Serial data on kidney lesion size of patients initiated on everolimus treatment were excluded from these analyses.

### Variable definition

Hypertension was defined as clinic systolic and/or diastolic blood pressure equal to or higher than the 95th percentile for sex-, age-, and height-matched children [13]. Due to incomplete data records or lack of repeated blood pressure measurements for some patients, any single finding indicative of hypertension was considered a case of hypertension. Furthermore, patients who were receiving antihypertensive treatment, even if their blood pressure readings were not recorded, were also considered hypertensive. Nephromegaly was defined as kidney length more than two standard deviations (SD) above the mean size of normal age-matched kidneys. Standard deviation values were obtained using the Paediatric Kidney Size Percentile Calculator [14]. Kidney function was assessed using the estimated glomerular filtration rate (eGFR), calculated using the Schwartz formula [eGFR = 36.5 × height (cm)/creatinine (µmol/L)] [15]. Note that a widely accepted definition of hyperfiltration is currently lacking, and estimating eGFR is not validated to determine hyperfiltration due to its age-dependent nature [16]. For this study, an age-unadjusted threshold of eGFR ≥ 140 mL/min/1.73 m^2^ was considered indicative of glomerular hyperfiltration [17]. On the other hand, we defined CKD to be from stages II–V, with eGFR < 90 mL/min/1.73 m^2^. Kidney intervention was defined as arterial embolisation or nephrectomy.

### Statistical analysis

Cohort characteristics were presented using descriptive statistics. Continuous variables were expressed as the mean with SD for normally distributed data and median with interquartile range for skewed data. Categorical variables were described using frequencies and percentages. The association between variables was analysed using the Pearson chi-square test or Fisher’s exact test for two categorical variables and the independent *t*-test for one continuous and one categoric variable. Survival probabilities were estimated with the Kaplan‒Meier curves, and the log-rank (Mantel‒Cox) test was conducted to assess the differences in survival curves among genetic mutations. Patients with no events (kidney lesions) were censored at the time of last imaging; right censoring therefore extends the survival analyses beyond the follow-up period in the study. Analyses were conducted using IBM SPSS Statistics 28. All statistical tests were two-sided and *p* values < 0.05 were considered statistically significant.

## Results

### Sociodemographic characteristics of participants

A total of 182 paediatric patients with TSC with a median age of 11 years were included in the analysis. Brain MRI was the modality used most frequently in the initial suspicion for TSC as performed in 137 patients (75.3%), followed by echocardiography in 12.6% (23/182) and CT scan and ultrasound in 9 (4.9%) and 3 (1.6%) patients, respectively. TSC was diagnosed at a median age of 8 months, 11 cases received a prenatal diagnosis, and one case was diagnosed at 16 years. By 2 years, three-quarters of children had been diagnosed, and only 5% (9/182) of children were diagnosed after 7 years.

Of all cases, 73.6% (134/182) of patients had genetic testing performed, and the results were *TSC1* 25/134 (18.7%), *TSC2* 93/134 (69.4%), *TSC2/PKD1* 4/134 (3.0%), and no mutations identified 12/134 (6.6%). For subsequent analyses, patients with *TSC2/PKD1* mutations (*n* = 4) will be included in the *TSC2* group (now *n* = 97), considering that patients with *TSC2/PKD1* mutations (termed contiguous gene syndrome, CGS) have *TSC2* gene mutations in addition to *PKD1* mutations. Supplementary Table 1 summarises the baseline characteristics of all paediatric TSC cases in this study and compares the profiles of patients with *TSC1* and *TSC2* mutations. Notably, a family history of TSC was more likely in the *TSC1* group, compared to the *TSC2* group; but in both groups, less than 50% of the cohort had a family history of TSC. Neurologic, dermatologic, and kidney manifestations were frequently observed. Neuropsychiatric disorders were common, with intellectual or learning disability identified in 71 children (39%), autism spectrum disorder in 60 (33%), speech and language disorders in 33 (18.1%), and attention-deficit/hyperactivity disorder in 10 (5.5%). Additionally, our study also recorded several recurrent comorbidities, including asthma in 6 cases (3.3%), scoliosis in 5 (2.7%), and arrhythmias (Wolff–Parkinson–White syndrome and atrioventricular block) in 4 patients (2.2%).

## TSC-associated kidney disease in children

Only those with imaging performed were included to document kidney involvement in paediatric patients with TSC. A total of 145 of 182 patients (79.7%) with TSC had available kidney imaging data on record; of these, 114 (78.6%) yielded abnormal findings suggestive of TSC-associated kidney disease—either AMLs or cysts or both. RCC was not observed in any patient. A total of 447 imaging reports were retrieved from 145 children, comprising 105 (23.5%) MRI and 342 (76.5%) ultrasound reports. Seventy-two patients (49.7%) had repeated ultrasound scans, and 38.6% (56/145) had both ultrasound and MRI examinations. CT scan was not utilised in any patient. Given the predominant utilisation of ultrasound, only data derived from ultrasound readings were used for survival analyses and assessment of kidney lesion size changes to mitigate potential disparities arising from the use of multiple imaging modalities.

Of the 145 patients, 20 (13.8%) had *TSC1* mutations, 81 (55.9%) had *TSC2* mutations, 11 (7.6%) had no mutations identified, and the genetics of the remaining patients were not known. Genetic mutations were significantly associated with AML occurrence in TSC patients (*p* = 0.029); however, this was not observed for any kidney lesions (*p* = 0.059) or cysts (*p* = 0.209) (Supplementary Table 2). Gender was not associated with the development of kidney lesions. Further analysis, including only patients with known *TSC1* and *TSC2* mutations, revealed significant differences in the number and laterality of any kidney lesions and kidney AMLs between both groups (Table 1).

**Table 1:** Characteristics of kidney lesions by *TSC1* and *TSC2* mutations

Characteristics	*TSC1* mutation		*TSC2* mutation		*p* value^a^ (*TSC1* vs. *TSC2*)
	*n*	%	*n*	%	
**Any kidney lesions**					
No. of patients	12/20	60.0	66/81	81.5	**0.040**
Lesion laterality^b^					
Unilateral	6/12	50.0	5/65	7.7	** < 0.001**
Bilateral	6/12	50.0	60/65	92.3	
Gender					
Male	7/12	58.3	33/66	50.0	0.595
Female	5/12	41.7	33/66	50.0	
**Cysts**					
No. of patients	7/20	35.0	45/81	55.6	0.100
Lesion laterality^b^					
Unilateral	4/7	57.1	12/44	27.3	0.186
Bilateral	3/7	42.9	32/44	72.7	
Gender					
Male	5/7	71.4	24/45	53.3	0.370
Female	2/7	28.6	21/45	46.7	
**AMLs**					
No. of patients	7/20	35.0	52/81	64.2	**0.018**
Lesion laterality					
Unilateral	4/7	57.1	5/52	9.6	**0.001**
Bilateral	3/7	42.9	47/52	90.4	
Gender					
Male	4/7	57.1	24/52	46.2	0.585
Female	3/7	42.9	28/52	53.8	

*AMLs* angiomyolipomas

^a^*p* value is obtained using chi-square test or Fisher’s exact test

^b^The laterality of cysts in one patient with *TSC2* mutations is not known

Seventy-eight of 145 (53.8%) patients displayed cysts on imaging, and 17 of these were unilateral—41.2% (7/17) were on the right kidney and 58.8% (10/17) on the left. Similarly, of the 88 patients (60.9%) with AMLs seen on imaging, only 11 had unilateral AMLs, predominantly in the left kidneys (72.7%, 8/11). Considering all 145 patients with imaging, there was a relatively equal distribution of cysts and AMLs on either side of the kidney; however, the predilection for the largest lesion for both cysts and AMLs was in the upper pole of either kidney.

**Fig. 1:**
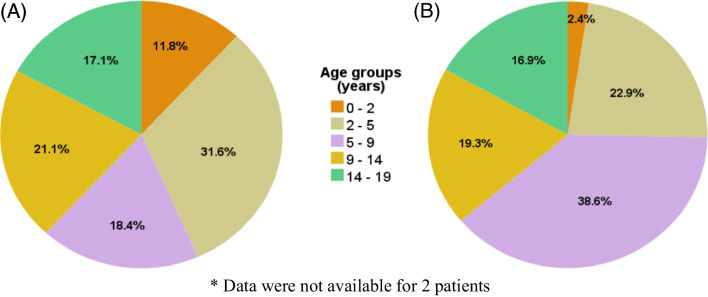
Age at first detection of TSC-associated kidney lesions on imaging; **A** kidney cysts (*N* = 78), **B** kidney AMLs (*N* = 88)

Figure 1 illustrates the age at which cysts and AMLs were first detected on kidney imaging. A total of 43.4% of children had cysts identified within the first 5 years of life. In contrast, over a third of AMLs were initially detected on imaging in children aged 5–9 years, with only 2.4% (2/88) of cases being in children under the age of 2 years.

### Time-to-event (survival) analyses of kidney lesions by genetic mutation

An estimation of the probability of being lesion-free with increasing age using the Kaplan–Meier method was performed. The result was then compared after stratifying according to genetic mutation using the log-rank test. Kidney lesion survival data were not available for 5 patients in the *TSC2* category. Sensitivity analysis was performed by repeating the analysis using a worst-case and best-case scenario, assuming the 5 missing data as either exhibiting lesion on day 0 (worst-case) or still lesion-free at their present age (best-case). The analysis showed that the results for kidney AMLs were not sensitive to missing data, but kidney cysts would be significantly affected by the missing data assuming the 5 children developed cysts since day 0 (*p* = 0.047). Across both mutations, the pooled median times for kidney cyst and AML development were 10.3 years and 8.2 years, respectively (Table 2).

**Table 2:** Time-to-event among patients with *TSC1* and *TSC2* mutations

Genetic mutation	No. in cohort	Events		Age range at initial detection (years)	Median time-to-event time (years)	Standard error	95% CI	*p* value (log-rank)
		*n*	%					
Kidney cysts								
*TSC1*	20	7	35.0	4.8–16.9	16.9	0.000	-	0.099
*TSC2*	76	44	57.9	0.1–17.6	9.1	2.042	5.081, 13.085	
Overall	96	51	53.1	0.1–17.6	10.3	1.860	6.688, 13.979	
Kidney AMLs								
*TSC1*	20	7	35.0	2.5–12.7	12.7	2.786	7.206, 18.127	**0.030**
*TSC2*	76	49	64.5	1.2–17.9	7.8	0.507	6.840, 8.827	
Overall	96	56	58.3	1.2–17.9	8.2	0.600	6.992, 9.342	

*AMLs* angiomyolipoma, *CI* confidence intervals

**Fig. 2:**
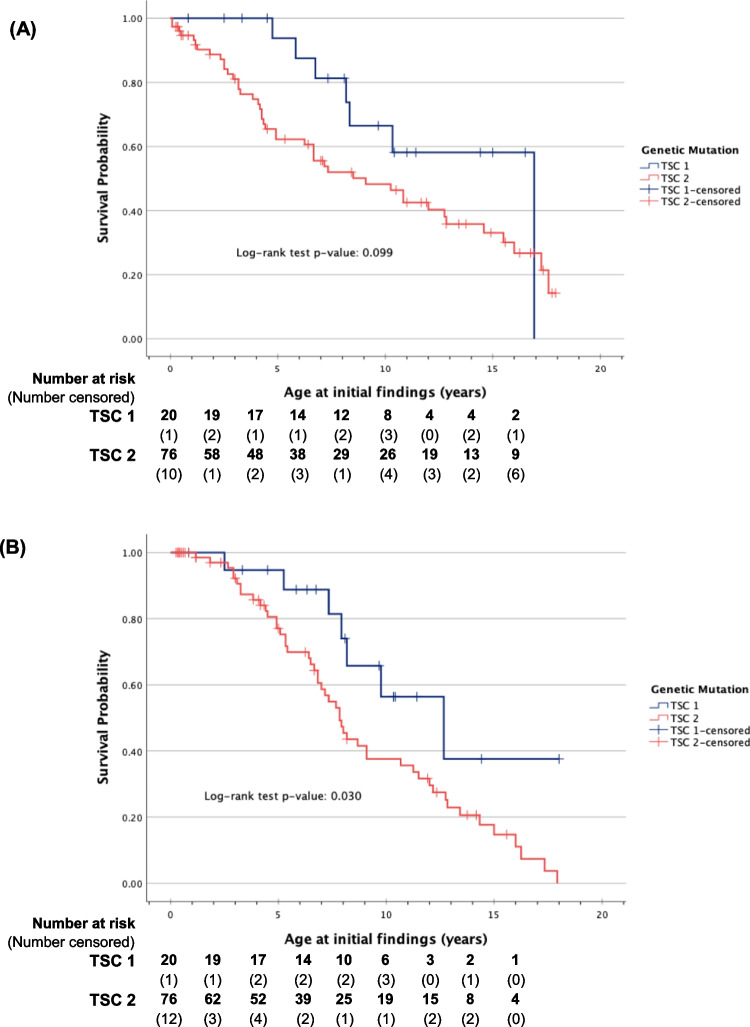
Kaplan–Meier curve in TSC paediatric patients for **A** kidney cysts and **B** kidney AMLs

Patients with *TSC1* mutations generally developed kidney lesions later than those with *TSC2* mutations (Fig. 2A). Kidney cysts in patients with *TSC1* mutations developed almost twice as late (16.9 vs. 9.1 years); however, there was no statistically significant difference in the time-to-event probability between the two cohorts. Figure 2B illustrates that kidney AML-free survival at 7 years was significantly better in the *TSC1* variant population (11/20, 55%) than in those with *TSC2* variants (26/76, 34.2%) (*p* = 0.030, log-rank test).^1^

### Kidney lesion growth

**Fig. 3:**
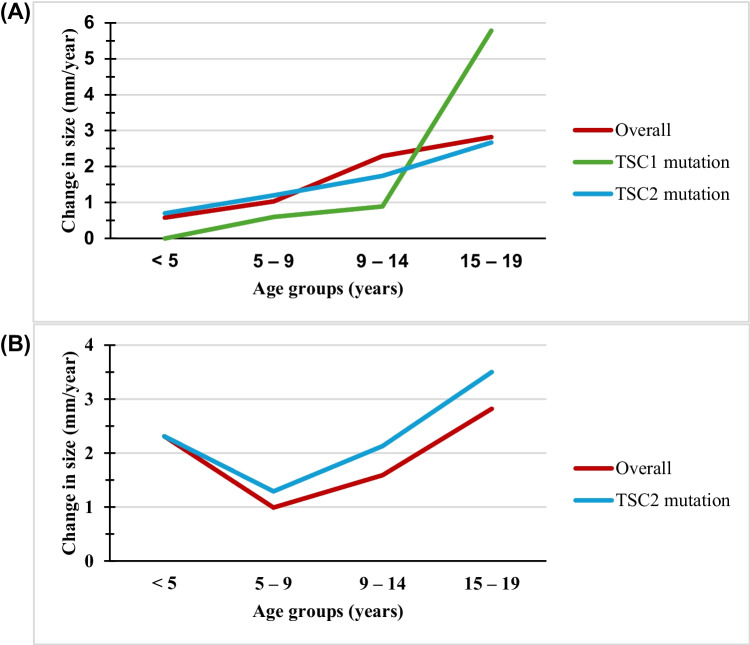
Change in the size of the largest **A** AMLs and **B** cysts in TSC paediatric patients by age group over time. There was no line for *TSC1* mutation in kidney cysts due to insufficient data in this cohort

Data on the size of kidney cysts and AMLs were retrieved from imaging reports, and each report was analysed as an independent measurement and then categorised by age groups. The change in the size of the largest AML accelerates with increasing age, from 0.58 mm/year in children under five to 2.82 mm/year in adolescents aged 14–19 years at the time of imaging (Fig. 3A, Table 3). We further attempted to analyse these data by genetic mutations and found similar conclusions. Figure 3B and Table 4 show the change in the size of the largest kidney cyst. While similar patterns were observed for kidney cysts from children above 5 years, we also found children under 5 to have relatively higher rates comparable to those of children aged 15 years (2.31 vs. 2.82 mm/year), although most data were from children with *TSC2* mutations.

**Table 3:** Change in the size of the largest kidney AMLs in TSC patients by age group over time

Age groups (years)	No. of data	Overall change (mm/year)	*TSC1* mutation			*TSC2* mutation		
			No. of data	Size change (mm/year)	Median size (IQR) in mm	No. of data	Size change (mm/year)	Median size (IQR) in mm
< 5	6	0.58	0	-	3.5 (3–4)	5	0.70	6.5 (4–10)
5–9	51	1.03	4	0.60	5 (2–5)	38	1.20	8 (5–13)
9–14	52	2.29	9	0.89	4.5 (2.5–9)	24	1.74	10 (7–17)
15–19	20	2.82	4	5.78	30 (12–40)	11	2.67	20 (9–28)

*IQR* interquartile range

**Table 4:** Change in the size of the largest kidney cysts in TSC patients by age group over time

Age groups (years)	No. of data	Overall change (mm/year)	*TSC1* mutation			*TSC2* mutation		
			No. of data	Size change (mm/year)	Median size (IQR) in mm	No. of data	Size change (mm/year)	Median size (IQR) in mm
< 5	26	2.31	0	-	1 (1–3)	26	2.31	8 (4–24)
5–9	33	0.99	0	-	3 (2–4)	21	1.29	8.5 (5–28)
9–14	25	1.59	0	-	6 (3–8)	18	2.13	7 (4–36)
15–19	9	2.82	1	0.9	5 (3–9)	7	3.50	9.5 (8–42)

*IQR* interquartile range

#### Complications and management of kidney lesions

Table 5 highlights kidney abnormalities and the proportion of children receiving therapy in our cohort. A total of 105 of 145 children (72.4%) had kidney function tested, but an eGFR estimate could not be derived from 24 due to missing height data. Of the remaining 81 children, 48.1% (39/81) had at least one episode of hyperfiltration, and 12.3% (10/81) showed kidney insufficiency when eGFR was determined using the bedside Schwartz formula. Analysis of kidney function abnormalities between *TSC1* and *TSC2* mutations yielded no statistical significance (Supplementary Table 3). Furthermore, our study found no association between kidney lesions and hypertension, nephromegaly, hyperfiltration, or CKD (Supplementary Table 4). However, subgroup analysis revealed a significant association between high-risk AMLs and nephromegaly, hyperfiltration, and CKD, but not hypertension (Supplementary Table 5). Stratification by age groups demonstrated that only hyperfiltration significantly differed between age groups (*p* = 0.035) (Supplementary Table 6). Of note, less than 10% of our patients received antihypertensive drugs, everolimus, or kidney interventions such as nephrectomy or embolisation.

**Table 5:** Kidney abnormalities and management

Parameters	Overall	
	*n*	%
Hyperfiltration (eGFR ≥ 140 mL/min/1.73 m^2^)^a^	39/81	48.1
Nephromegaly (> 2 SD)^b^	28/85	32.9
Hypertension (≥ 95th centile)^c^	31/125	24.8
Chronic kidney disease (eGFR < 90 mL/min/1.73 m^2^)	10/81	12.3
Kidney AMLs ≥ 3 cm (high-risk)	13/145	9.0
On antihypertensives	9/125	7.2
On everolimus	13/182	7.1
Kidney interventions	3/182	1.6

*AMLs* angiomyolipomas, *eGFR* estimated glomerular filtration rate, *SD* standard deviation

^a^eGFR measurement was calculated using GFR calculator [38]

^b^Kidney size was determined using Pediatric Kidney Size Percentile Calculator [14]

^c^Blood pressure centile was obtained using MDCalc [39]

## Discussion

Tuberous sclerosis is a heterogeneous disorder that typically presents during infancy. Our research revealed that the median age of TSC diagnosis was 8 months, aligning with the multi-country TOSCA study involving 2211 TSC patients spanning all ages [18]. *TSC2* mutations were more prevalent than *TSC1* mutations, with a ratio of 3.7 to 1, in line with previous studies [18, 19]. However, it should be acknowledged that mutation analysis was not routinely conducted in 26.4% (48/182) of children in our study. Nair and colleagues [20] highlighted that approximately 70% of TSC cases arise from de novo mutations; our study found 80.2%. Our study demonstrated higher organ involvement in patients with *TSC2* mutations than *TSC1* mutations, consistent with earlier findings that *TSC2* is associated with more severe disease characteristics [21]. Back pain attributed to kidney lesions in TSC patients is an uncommon presentation and has rarely been reported in previous studies, but our study identified five children (2.7%) who presented with back pain. All the cases were in children above 8 years of age and had nephromegaly, though they presented with varying kidney phenotypes.

Detecting kidney involvement in TSC necessitates imaging to identify lesions, as small lesions may be incidental findings, and most patients with TSC kidney disease are asymptomatic. MRI is the preferred imaging modality for diagnosis and follow-up of TSC-related kidney lesions stemming from its superior soft tissue resolution and multiplanar capabilities without radiation exposure [22]; however, despite its recommendation in the latest consensus [22, 23], MRI was used in only 23.5% of cases in our study. The limited utilisation of MRI in our cohort can be attributed to several factors. MRI is primarily chosen for the evaluation of large AMLs to monitor growth and complications and MRI often necessitates sedation in younger children and may be avoided by caregivers of children with TSC [22]. An alternative imaging modality is ultrasound, accounting for over 75% of the imaging used in our study. However, it should be noted that the quality of ultrasound imaging is operator-dependent and may miss sizable lipid-poor masses (such as AMLs) and echogenic kidney foci that are too small to characterise into either AMLs or cysts [11, 24]. Moreover, conducting ultrasound scans on children with TSC can be challenging. The frequency of kidney assessment should be individualised, with annual follow-ups deemed sufficient for stable or lesion-free patients [23, 25]. Considering differences in disease progression between TSC phenotypes, we recommend genetic testing to be performed in all patients with suspicion of TSC, and more frequent monitoring of kidney function and imaging (at least annually) is advised for older children, with an earlier age threshold for children with *TSC2* mutation. Current recommendations advocate for CT use only if MRI is unavailable, with CT angiography reserved for AMLs above 3 cm to exclude intra-lesional microaneurysms or pinpoint bleeding sources in haemorrhagic cases, despite weak evidence supporting this recommendation [8, 25]. However, no CT scans were conducted in our study due to concerns about radiation risk.

Among the 145 examined children, kidney imaging revealed lesions in 114 (78.6%), comprising cysts (53.8%), AMLs (61.1%), or both (45.6%). This concurs with Bissler et al.’s [5] recent review on kidney involvement. While kidney cysts were slightly more prevalent in males and AMLs in females, we did not find a significant association between gender and lesion prevalence, consistent with other reports [26, 27]. The TSC genotype is a predictor for kidney involvement [20], as supported by our results showing higher incidences of both cysts and AMLs in patients with *TSC2* mutations compared to *TSC1*; however, this significance was noted only for AMLs, not for cysts. Our results, echoing Kingswood et al. [18], highlighted significantly higher rates of bilateral AMLs in *TSC2* patients than in *TSC1* patients. This finding aligns with data indicating multiple and bilateral distributions of most AMLs and cysts [11, 26]. Interestingly, we observed that the largest AMLs were primarily in the left kidney (72.7%). Kidney lesion laterality has not been investigated extensively, but previous studies on RCC have also found that patients with left-sided lesions had poorer outcomes than those with right-sided RCC [28], suggesting that lesion laterality in kidneys may not be merely coincidental. Kidneys differ in anatomy, vascular supply, and lymphatic drainage between the left and right kidneys; the left kidney has more vascular collateral circulation and lymph nodes. Cutaneous TSC lesions demonstrated proliferation and dilatation of lymphatic and blood vessels on histology [29]; as such, this increased vascularity may deliver more vascular endothelial growth factors necessary for angiogenesis and lymphangiogenesis, inducing the development of larger AMLs found in the left kidney.

Cook et al. [30] suggested that cysts were more common than AMLs in children under 5. Our study found that over 40% of children under 5 had cysts, compared to approximately 25% with AMLs. Note that we were unable to exclude the possibility that patients may have already had kidney cysts or AMLs prior to the initial imaging episode. Kidney AMLs are relatively infrequent in children under 2, likely due to their slow growth and small size, often missed on imaging. Our data show that the change in the size of the largest AML accelerates with age: 1.03 mm/year (ages 5–9 years), 2.29 mm/year (ages 9–14 years), and 2.82 mm/year (ages 15–19 years). Robert and colleagues [31] also observed AML growth doubling before and after 12 years (from 2 mm/year to 4.5 mm/year), though their observation was based on data from 21 patients with AML, and the authors did not specify how these rates were obtained. Interestingly, Ewalt et al. [32] reported a case of an 18-year-old man with AML growth at 4 cm per year, although they did not provide further details about this case, including the genetic makeup. The influence of oestrogen and progesterone may explain this surge, given the hormonal receptors on the tumour surface [33]. As such, peri-pubertal, pregnancy, or hormonal treatment phases could significantly boost AML growth due to the enhancing effects of these hormones. Also, while ultrasound may be too insensitive for accurate measurements of kidney lesions, MRI also faces limitations as the precision in measurements depends on its slice periodicity (i.e. growth less than a single slice distance), and thus inaccuracy in measuring smaller lesions is possible. Prospective studies involving expert radiologists are necessary to accurately measure and calculate the longitudinal growth rates of individual lesions, aiding in determining the recommended interval for follow-up.

To the best of our knowledge, only a Belgian study, conducted by Janssens et al., has explored cyst growth in TSC cases [7]. They observed a median cyst growth of 0.2 mm/year among 45 patients; however, the researchers did not conduct any further analysis based on age or genetic mutations. Our study yielded novel findings, with kidney cysts displaying two peaks: the first in children under five and another between 15 and 19 years. We acknowledge that our findings might have been confounded by including four patients with CGS, where CGS patients are known to experience more severe polycystic kidney growth and earlier onset of kidney impairment [8]. Even after the exclusion of these four cases from our analysis, a similar pattern persisted. Although we could not find existing literature explaining these specific findings, we hypothesise that a combination of factors might contribute to increased cyst growth during the first 5 years of life. These factors include heightened cellular proliferation, a rapid kidney growth rate (especially in the early years) [34], and, to a lesser extent, an altered hormonal milieu in early childhood [35].

In our analysis, we observed variations in the time to cyst development between children with *TSC1* and *TSC2* mutations, albeit the difference did not reach statistical significance, likely due to the study being underpowered to detect kidney cysts from the limited *TSC1* cases (*n* = 20). Nevertheless, *TSC1* demonstrated better cyst-free survival (median 16.9 years) than *TSC2* (median 9.1 years) at any time. On another note, our study underscores the importance of conducting dedicated studies focused solely on children with TSC. For instance, the TOSCA study that included patients across all ages revealed the mean age at AML diagnosis of 22.5 years for *TSC1* and 13.3 years for *TSC2* [18]. In contrast, our study observed a median age at AML development of 12.7 and 7.8 years, respectively. These disparities in the age of kidney lesion onset highlight the imperative of acknowledging such variations when formulating clinical practice guidelines for managing these cases.

While most patients with AMLs remain asymptomatic [25], those that exceed 3 cm in size are associated with an increased risk of haemorrhage—the main complication of AML and a leading cause of mortality in TSC [27, 36]. In our study, we observed that 9% (13/145) of children developed AMLs above 3 cm, with the majority exhibiting *TSC2* mutations. The median age at which AML size exceeds 3 cm was 13.8 years (range: 5.3–18.9 years). Subsequent follow-up primarily utilised MRI. Notably, more than half of these patients (7/13) experienced AML growth beyond 4 cm, with only four of them receiving everolimus treatment. Current clinical guidelines suggest that individuals with AMLs exceeding 3 cm may benefit from everolimus treatment, as shown to hold therapeutic potential in the EXIST-1 to EXIST-3 trials [22, 23, 36, 37]. However, other factors, including physician expertise and clinical judgement, may influence the decision to initiate everolimus treatment. Among the remaining nine children receiving everolimus treatment for neurologic indications, none developed high-risk kidney lesions, and we lacked sufficient data to determine the effect of everolimus on AMLs of relatively small size. Nonetheless, none of the patients with high-risk AMLs experienced acute bleeding. Apart from haemorrhage, TSC kidney lesions can progressively replace functional tissue, leading to early GFR impairment and secondary hypertension. Our study observed AMLs exceeding 3 cm were significantly associated with developing nephromegaly (*p* = 0.028), hyperfiltration (*p* = 0.014), CKD (*p* = 0.001), and being initiated on everolimus treatment (*p* = 0.007) (see Supplementary Table 4).

In our study, three (1.6%) of 182 children with TSC underwent kidney interventions, including partial nephrectomies in 2 children and embolisation in 1. The two cases of partial nephrectomy were performed in children aged 8 and 14 years. In one case nephrectomy was indicated due to nephromegaly (right kidney > 21 cm). Neither hypertension nor other complications were detected pre- and post-nephrectomy in both cases. Only one patient underwent embolisation due to AMLs measuring over 5 cm, without acute bleeding. Our findings are consistent with the TOSCA study, which suggests that everolimus usage is more common than embolisation and nephrectomy combined [12].

Hyperfiltration, hypertension, and proteinuria are known risk factors for kidney disease progression [7]. Although these factors were prevalent in our study, we did not find a significant association with CKD, similar to the findings of the Belgian study [7]. It is worth noting that the definition of glomerular hyperfiltration based on eGFR is still lacking and not validated, and thus may affect its accuracy in capturing the true incidence of hyperfiltration [16]. Out of 81 children, thirty-nine (48.1%) experienced at least one episode of hyperfiltration. Although overestimation is possible due to methodological constraints, and some patients lacked longitudinal kidney function data on record, evidence suggests that hyperfiltration was transient in some children. Furthermore, a third of the cases with a hyperfiltration episode were detected in children under the age of 5 years, supporting the recommendation that at least annual biochemical testing to monitor kidney function should be performed in children with proven kidney involvement on imaging [22]. CKD, which we defined as stages II–V and eGFR below 90 mL/min/1.73 m^2^, was identified in 10 children in our study: 8 children were at stage II, 1 child at stage III (eGFR < 60), and 1 child at stage IV (eGFR < 30). Both cases with advanced CKD (stage III–V) had *TSC2/PKD1* mutation. The child with stage III CKD (aged 5 years) had developed multiple small kidney cysts and multiple AMLs, with two AMLs measuring above 4 cm with multiple antihypertensive drugs. The other case with CKD stage IV (aged 16 years) had an eGFR of 29 and developed bilateral, multiple, and large cysts on both kidneys. No other potential cause of CKD was identified in this case. Mekahli et al. [22] highlighted that the precise proportion of TSC patients with CKD remains unclear, partly due to the absence of TSC as a diagnosis code in kidney failure databases. Regarding other variables analysed, such as hypertension or being on everolimus, no significant associations were observed with the presence of kidney lesions, high-risk AMLs, or even after stratification by age groups. This lack of significant findings is likely attributable to the study being underpowered to determine meaningful predictions.

Limitations of this study include the retrospective nature resulting in varied follow-up times for patients, potentially leading to an underestimation of kidney lesions or complications in some cases. The exclusion of patients without imaging reports may introduce bias, and reliance on reports from multiple radiologists may lead to imprecise measurements and difficulty distinguishing disease progression from previously missed lesions. Missing data in patient records could also distort our findings or result in underpowered analyses; however, our sensitivity analyses revealed significance only for cyst-free survival. Our study did not longitudinally track the serial dimensions of kidney lesions, but instead data were collected in a cross-sectional manner with only the largest lesion noted at each time point. Consequently, this may potentially lead to an underestimation of lesion growth.

In conclusion, this study offers valuable insights from 145 children with tuberous sclerosis and kidney imaging data available, which is a substantial cohort given the rarity of the condition. Novel findings in this study include the location of kidney lesions, kidney cyst growth, and survival analyses for both kidney cysts and AMLs. While TSC kidney disease emerges later in the disease course than neurological features, routine and effective surveillance of the incidence and growth velocity of cysts and AMLs and their progression, including regular kidney imaging, kidney function, and blood pressure monitoring, should be adopted for these patients during childhood.

### Supplementary Information

Below is the link to the electronic supplementary material.

Graphical abstract (PPTX 312 KB)

Supplementary file2 (DOCX 33 KB)

Supplementary file3 (DOCX 38 KB)

## Data Availability

The datasets generated during and/or analysed during the current study are available from the corresponding author on reasonable request.

## Footnotes

1 The inclusion of 4 cases of *TSC2/PKD1* mutations within the *TSC2* cohort may potentially distort the true findings, particularly in regard to survival analysis of kidney cysts. Thus, we attempted to re-analyse the data by excluding the 4 cases (now *TSC2* cohort *n* = 93). The kidney phenotypes of these children varied: 2 patients developed cysts only, 1 patient had normal imaging up to 20 years of age, and the remaining patient had no imaging on record. Upon conducting survival analyses, we found no difference in the median time-to-event time for both cysts and AML. While the *p*-value for log-rank test changed, the survival time for AML between *TSC1* and *TSC2* mutation remain significantly different (*p* = 0.027), whereas kidney cyst remained not significant (*p* = 0.125).

## Abbreviations

*AML*: Angiomyolipoma
*CGS*: Contiguous gene syndrome (i.e. *TSC2/PKD1* mutation)
*CKD*: Chronic kidney disease
*eGFR*: Estimated glomerular filtration rate
*RCC*: Renal cell carcinoma
*SD*: Standard deviation
*TSC*: Tuberous sclerosis complex
