# Supplementary material for: The extent of kidney involvement in paediatric tuberous sclerosis complex
Source: Pediatr Nephrol. 2024 Jun 4;39(10):2927–37. doi: 10.1007/s00467-024-06417-2 (PMC11349837; doi:10.1007/s00467-024-06417-2)
Supplement: Supplementary file 1 — Graphical abstract (PPTX 312 KB) [file 467_2024_6417_MOESM1_ESM.pptx]

## Slide 1
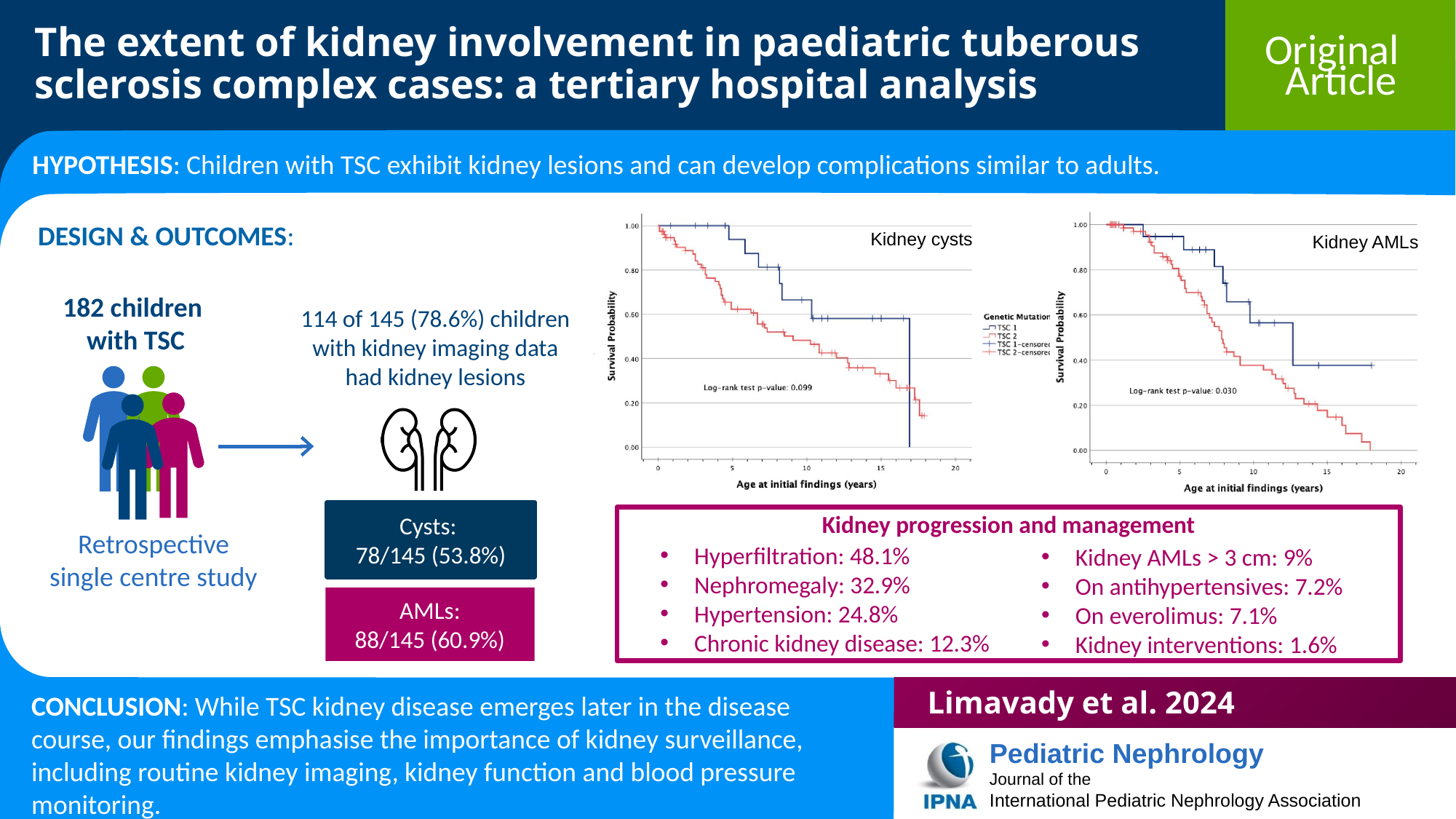

The extent of kidney involvement in paediatric tuberous
sclerosis complex cases: a tertiary hospital analysis
HYPOTHESIS: Children with TSC exhibit kidney lesions and can develop complications similar to adults.
Kidney cysts
Kidney AMLs
DESIGN & OUTCOMES:
182 children
with TSC
114 of 145 (78.6%) children with kidney imaging data had kidney lesions
Cysts:
78/145 (53.8%)
Kidney progression and management
Retrospective
single centre study
Hyperfiltration: 48.1%
Nephromegaly: 32.9%
Hypertension: 24.8%
Chronic kidney disease: 12.3%
Kidney AMLs > 3 cm: 9%
On antihypertensives: 7.2%
On everolimus: 7.1%
Kidney interventions: 1.6%
AMLs:
88/145 (60.9%)
Limavady et al. 2024
CONCLUSION: While TSC kidney disease emerges later in the disease course, our findings emphasise the importance of kidney surveillance, including routine kidney imaging, kidney function and blood pressure monitoring.
