# Supplementary material for: The extent of kidney involvement in paediatric tuberous sclerosis complex
Source: Pediatr Nephrol. 2024 Jun 4;39(10):2927–37. doi: 10.1007/s00467-024-06417-2 (PMC11349837; doi:10.1007/s00467-024-06417-2)
Supplement: Supplementary file 3 — Supplementary file3 (DOCX 38 KB) [file 467_2024_6417_MOESM3_ESM.docx]

**Supplementary Data**

**Supplementary Table 1**. Clinical profiles of paediatric patients with TSC.

|  | **Overall**  N = 182 | | ***TSC1* mutation**  N = 25 | | ***TSC2* mutation**  N = 97 | | ***p value* ^a^**  (*TSC1* vs. *TSC2*) |
| --- | --- | --- | --- | --- | --- | --- | --- |
|  | **n** | **%** | **n** | **%** | **n** | **%** |  |
| **Median age (IQR),** years | 11 (6.7–16.3) | | 10.4 (6.9–14.6) | | 9.9 (5.5–15.8) | | 0.656 |
| **Gender** | | | | | | | |
| Male | 94 | 51.6 | 15 | 60.0 | 52 | 53.6 | 0.567 |
| Female | 88 | 48.4 | 10 | 40.0 | 45 | 46.4 |  |
| **Family history of TSC** | | | | | | | |
| Yes | 36 | 19.8 | 12 | 48.0 | 14 | 14.4 | **< 0.001** |
| No | 146 | 80.2 | 13 | 52.0 | 83 | 85.6 |  |
| **Symptoms at diagnosis** | | | | | | | |
| Seizure | 171 | 94.0 | 22 | 88.0 | 95 | 97.9 | **0.025** |
| Skin lesion or rash | 171 | 94.0 | 23 | 92.0 | 93 | 95.9 | 0.601 |
| Vision abnormalities | 52 | 28.6 | 8 | 32.0 | 31 | 32.0 | 0.997 |
| Back pain or haematuria | 5 | 2.7 | 0 | 0.0 | 4 | 4.1 | 0.580 |
| Headache | 5 | 2.7 | 1 | 4.0 | 1 | 1.0 | 0.369 |
| **Organ systems involvement** | | | | | | | |
| Brain lesions | 175 | 96.2 | 24 | 96.0 | 94 | 96.9 | 0.999 |
| Epilepsy (caused by brain tumour) | 172 | 94.5 | 23 | 92.0 | 95 | 97.9 | 0.186 |
| Hypomelanotic macule | 162 | 89.0 | 21 | 84.0 | 88 | 90.7 | 0.465 |
| Neuropsychiatric disorder | 140 | 76.9 | 14 | 56.0 | 81 | 83.5 | **0.003** |
| Kidney lesions | 114 | 62.6 | 12 | 48.0 | 66 | 68.0 | 0.063 |
| Heart rhabdomyoma | 83 | 45.6 | 11 | 44.0 | 54 | 55.7 | 0.297 |
| Facial angiofibroma | 81 | 44.5 | 8 | 32.0 | 40 | 43.0 | 0.320 |
| Eye lesions | 26 | 14.3 | 1 | 4.0 | 20 | 20.6 | 0.072 |

Abbreviations: IQR, interquartile range; TSC, Tuberous Sclerosis Complex

^a^ p value was obtained using independent t test, Chi-squared test, and Fisher’s exact test.

**Supplementary Table 2.** Lesion characteristics in patients with kidney imaging.

|  | **Any kidney lesions** | | **Cysts** | | | **AMLs** | | |
| --- | --- | --- | --- | --- | --- | --- | --- | --- |
|  | n | % | n | % | | n | % | |
| No. of patients | 114/145 | 78.6 | 78/145 | 53.8 | | 88/145 | 61.1 | |
| **Laterality** | | | | | | | | |
| Unilateral | 13/114 | 11.4 | 17/78 | 21.8 | | 11/88 | 12.5 | |
| Bilateral | 71/114 | 62.3 | 39/78 | 50.0 | | 53/88 | 60.2 | |
| Not known | 30/114 | 26.3 | 22/78 | 28.2 | | 24/88 | 27.3 | |
| **Gender** | | | | | | | | |
| Male | 58/114 | 50.9 | 42/78 | 53.8 | | 42/88 | 47.7 | |
| Female | 56/114 | 49.1 | 36/78 | 46.2 | | 46/88 | 52.3 | |
| OR (95% CI) ^a^ | 1.030 (0.465, 2.279) | | 0.784 (0.407, 1.507) | | | 1.402 (0.717, 2.739) | | |
| *p value* ^b^ | 0.942 | | 0.465 | | | 0.322 | | |
| **Genetic mutations** | | | | | | | | |
| *TSC1* | 12/114 | 10.5 | 7/78 | | 9.0 | 7/88 | | 8.0 |
| *TSC2* | 66/114 | 57.9 | 45/78 | | 57.7 | 52/88 | | 59.1 |
| NMI | 7/114 | 6.1 | 5/78 | | 6.4 | 5/88 | | 5.7 |
| Not known | 29/114 | 25.4 | 21/78 | | 26.9 | 24/88 | | 27.3 |
| *p value* ^b^ | 0.053 | | 0.209 | | | **0.029** | | |

Abbreviations: AMLs, angiomyolipomas; CI, confidence intervals; NMI, no mutation identified; OR, odds ratio

^a^ female as the reference category for odds ratios.

^b^ *p value* is obtained using Chi-square test or Fisher’s exact test.

**Supplementary Table 3**. Association of kidney abnormalities and genetic mutations

| **Characteristics** | ***TSC1* mutation**  n (%) | ***TSC2* mutation**  n (%) | **Odds ratio**  (95% CI) | ***p value* ^a^** |
| --- | --- | --- | --- | --- |
| AMLs > 3 cm | 1/20 (5) | 8/81 (9.9) | 2.082  (0.24–17.684) | 0.684 |
| Hypertension (≥ 95^th^ centile) | 5/16 (31.3) | 16/62 (25.8) | 0.765  (0.230–2.542) | 0.662 |
| Nephromegaly (> 2 SD) | 1/8 (12.5) | 18/51 (35.3) | 3.818  (0.435–33.527) | 0.200 |
| Hyperfiltration (eGFR ≥ 140 mL/min/1.73 m^2^) | 3/10 (30) | 26/45 (57.8) | 3.193  (0.730–13.974) | 0.164 |
| Chronic kidney disease (eGFR < 90 mL/min/1.73 m^2^) | 0/10 (0) | 6/45 (13.3) | - | 0.579 |

Abbreviations: AMLs, angiomyolipomas; CI, confidence intervals; eGFR, estimated glomerular filtration rate; SD, standard deviation

^a^ *p value* is obtained with Chi-square test or Fisher’s exact test.

**Supplementary Table 4**. Association of kidney abnormalities and the presence of lesions

| **Characteristics** | **Kidney lesions** | | ***p value*** ^a^ |
| --- | --- | --- | --- |
|  | **Yes**  n (%) | **No**  n (%) |  |
| Hypertension (≥ 95^th^ centile) | 25/89 (28.1) | 6/36 (16.7) | 0.181 |
| Nephromegaly (> 2 SD) | 27/77 (35.1) | 1/8 (12.5) | 0.196 |
| Hyperfiltration (eGFR ≥ 140 mL/min/1.73 m^2^) | 33/68 (48.5) | 6/13 (46.2%) | 0.875 |
| Chronic kidney disease (eGFR < 90 mL/min/1.73 m^2^) | 5/46 (10.9) | 1/9 (11.1) | 0.983 |
| On antihypertensives | 7/89 (7.9) | 2/36 (5.6) | 0.999 |
| On everolimus | 11/114 (9.6) | 2/68 (2.9) | 0.136 |
| Kidney interventions | 3/114 (2.6) | 0/68 (0) | 0.294 |

^a^ *p value* obtained with Chi-square test or Fisher’s exact test.

**Supplementary Table 5**. Association of kidney abnormalities and AML > 3 cm

| **Characteristics** | **AMLs > 3 cm** | | ***p value* ^a^** |
| --- | --- | --- | --- |
|  | **Yes**  n (%) | **No**  n (%) |  |
| Hypertension (≥ 95^th^ centile) | 5/12 (41.7) | 20/76 (26.3) | 0.273 |
| Nephromegaly (> 2 SD) | 8/13 (61.5) | 19/64 (29.7) | **0.028** |
| Hyperfiltration (eGFR ≥ 140 mL/min/1.73 m^2^) | 1/9 (11.1) | 32/58 (55.2) | **0.014** |
| Chronic kidney disease (eGFR < 90 mL/min/1.73 m^2^) | 4/9 (44.4) | 4/58 (6.9) | **0.001** |
| On antihypertensives | 1/12 (8.3) | 6/76 (7.9) | 0.958 |
| On everolimus | 4/13 (30.8) | 7/100 (7) | **0.007** |
| Kidney interventions | 1/13 (7.7) | 2/100 (2) | 0.230 |

Abbreviations: AMLs, angiomyolipomas; eGFR, estimated glomerular filtration rate; SD, standard deviation

^a^ *p value* is obtained with Chi-square test or Fisher’s exact test.

**Supplementary Table 6**. Association of kidney abnormalities by age groups

| **Characteristics** | **Age groups** (years) | | | | ***p value*** ^a^ |
| --- | --- | --- | --- | --- | --- |
|  | **< 5**  n (%) | **5 ≤ 9**  n (%) | **9 ≤ 14**  n (%) | **≥ 14**  n (%) |  |
| Hypertension (≥ 95^th^ centile) | 4/7 (57.1) | 7/21 (33.3) | 4/20 (20) | 10/41 (24.4) | 0.247 |
| Nephromegaly (> 2 SD) | 1/6 (16.7) | 7/18 (38.9) | 6/19 (31.6) | 13/34 (38.2) | 0.739 |
| Hyperfiltration (eGFR ≥ 140 mL/min/1.73 m^2^) | 3/6 (50) | 7/14 (50) | 13/17 (76.5) | 10/31 (32.2) | **0.035** |
| Chronic kidney disease (eGFR < 90 mL/min/1.73 m^2^) | 0/6 (0) | 2/14 (14.3) | 1/17 (5.9) | 5/31 (16.1) | 0.568 |
| On antihypertensives | 2/7 (28.6) | 3/21 (14.3) | 0/20 (0) | 2/41 (4.9) | 0.056 |
| On everolimus | 0/10 (0) | 0/25 (0) | 4/28 (14.3) | 7/51 (13.7) | 0.145 |
| Kidney interventions | 0/10 (0) | 0/25 (0) | 1/28 (3.6) | 2/51 (3.9) | 0.712 |
| AML > 3 cm | 0/10 (0) | 2/24 (8.3) | 2/28 (7.1) | 9/51 (17.6) | 0.267 |

^a^ *p value* obtained with Chi-square test or Fisher’s exact test.
